# Defining compassion: A Delphi study of compassion therapists’ experiences when introducing patients to the term ‘compassion’

**DOI:** 10.1111/papt.12423

**Published:** 2022-08-24

**Authors:** Kirsten McEwan, Lina Minou

**Affiliations:** ^1^ Health, Psychology, and Social Care College Derby University Derby UK; ^2^ Philosophy Department University College London‐UCL London UK

**Keywords:** CFT, compassion, definition, Delphi, experiential, resistance

## Abstract

**Objectives:**

Compassion‐focused therapy (CFT) is shown to be an effective psychological intervention; however, patients can experience resistance to CFT due to preconceptions regarding the term ‘compassion’. This study aims to obtain guidance from therapists in how to overcome these resistances

**Design:**

This is the first study using the Delphi methodology to ask CFT therapists about how their patients understanding of the term compassion might act as a barrier to engaging with an otherwise beneficial therapy.

**Methods:**

Two rounds of interview questions were posed to 15 expert CFT therapists.

**Results:**

The results provide verification that there is resistance to CFT due to preconceptions around ‘compassion’, specifically its association with ‘pity’, ‘weakness’ and low‐rank social positions. Further, this appears to be pronounced in patients who value competitiveness.

**Conclusions:**

The results have practical implications such as the need for therapists to acknowledge the potential for resistance and the need for experiential strategies and illustrative examples of compassion to facilitate successful engagement with CFT.

**Practitioner Points**

- Resistance to compassion occurs in therapeutic settings and is connected to misconceptions of compassion as pity, weakness and ‘softness’.
- Valuing competitiveness and de‐valuing compassion influences the definition of and resistance to compassion.
- Defining compassion is insufficient and exposure to examples and experiences of compassion is necessary to reach clarification.
- Time emerged as an important factor in reaching an understanding of compassion.

## INTRODUCTION

In the treatment of mental health difficulties, many talking therapies (e.g. cognitive behavioural therapy) have focused on decreasing negative affect. Negative affect is part of a defensive response to threat which narrows attention and actions, allowing individuals to process and respond to threat (Richards et al., 2014). This narrowing of attention to deal with threats coincides with a shut‐down of positive affect, particularly low arousal positive effects associated with the social emotions of feeling the warmth and safety of others (McManus et al., 2019). However, these prosocial positive effects have a broadening effect on attention and can mitigate the downward spiral into mental health difficulties (Frederickson & Losada, 2005). Hence, instead of working to reduce negative effect, some therapeutic interventions (e.g. compassion‐focused therapy) focus on increasing prosocial positive effect. The ability to experience prosocial and low arousal positive effect can be increased through developing compassion.

Compassion emerged from the mammalian caregiving and care‐receiving system and is commonly defined as ‘a sensitivity to suffering in self and others, with a commitment to try to alleviate and prevent it’ (Gilbert, 2015, pp. 241). This definition complements wider compassion definitions discussed by Mascaro et al. (2020) in their review of how compassion is defined and measured, where they summarise, that compassion is a benevolent emotional response towards another who is suffering, coupled with the motivation to alleviate their suffering and promote their well‐being. Compassion is malleable, coinciding with structural changes in the neural networks associated with positive emotions and emotion regulation (Förster & Kanske, 2021). Exploiting the malleability of the capacity to develop compassion has been suggested to be a promising way to improve psychotherapeutic interventions and has become an important therapeutic goal (Förster & Kanske, 2021; Seppala, 2017). Several compassion‐based interventions have become available and reviews and meta‐analyses demonstrate their effectiveness in improving well‐being across both clinical and non‐clinical populations (Craig et al., 2020; Kirby, 2017; Leaviss & Uttley, 2015).

Although the therapeutic benefits of compassion‐focused therapy (CFT) are often supported by moderate effect sizes (Kirby et al., 2019), the process of developing compassion in clinical populations is challenging and often fears, blocks and resistances are experienced by patients, which sometimes lead to therapists opting for an alternative. Overcoming barriers to developing compassion is crucial to tackling some of the maintenance factors which often characterise mental health difficulties. For example, Kirby et al. (2019) revealed in a meta‐analysis that fears of self‐compassion and fears of receiving compassion from others were highly correlated with shame, self‐criticism and depression (Kirby et al., 2019).

This study therefore conducted a qualitative review (using the Delphi approach) with the pioneers and trainers of CFT to assess how they collaborated with patients to overcome fears blocks and resistances and develop compassion.

Fears of compassion are such a common barrier to patients' early engagement with CFT that a psychometric scale was developed to assess and monitor these fears (Gilbert et al., 2011). The items in the scale were generated in collaboration with patients who had received CFT and include fears of (1) indulging in pity, being selfish; (2) becoming weak or low‐ranking; (3) losing self‐criticism which was seen as essential in not becoming arrogant or lazy; (4) becoming overwhelmed with grief and sadness associated with one's own or another person's distress; (5) not deserving compassion. Clinicians and researchers have found that in particular, misconceptions around compassion being defined as pity or weakness can act as a key barrier to developing compassion for self and receiving it from others and could hinder engagement with compassion‐based interventions (de Zulueta, 2013; Gilbert et al., 2014; Steindl et al., 2022). It is remarked that these barriers come down to the language we use and the way we define compassion, bringing it close to ‘soft kindness, being nice, but also as weak and self‐indulgent, in a world context that demands toughness’ (Gilbert et al., 2019, pp. 2260). A further common fear of developing compassion comes from the fear of grief and overwhelm when engaging empathically with one's own or other people's distress. Singer and Klimecki (2014) noted that empathy can result in negative feelings and withdrawal, whilst in contrast compassion often results in positive, other‐oriented feelings and prosocial motivations. Indeed, the confusion of the terms compassion and empathy have led to the term ‘compassion fatigue’ being used in health care services, where empathic distress may actually be the cause of fatigue and withdrawal, whilst in contrast compassion facilitates resilience, well‐being and prosocial behaviours (Sinclair et al., 2017).

Given the challenges and complexities patients and practitioners face in overcoming fears of compassion, this study aimed to reach a consensus from expert practitioners about how patients understand the term ‘compassion’ throughout their therapeutic journey and gain an insight into recommendations for overcoming fears and working with individuals to develop compassion. Through an iterative process of obtaining expert opinions, called the ‘Delphi technique’, some of the most prominent CFT practitioners who pioneered CFT, are responsible for training hundreds of CFT therapists and have delivered CFT to great numbers of patients, provided their insights. Most of our respondents have been active in the development, research and implementation of CFT since its conception in 2006.

A recent study (Steindl et al., 2022) surveyed 64 CFT practitioners and asked them open‐ended questions about their recommendations for overcoming fears of compassion. This resulted in the following guidelines: (1) Fears of compassion are a part of the CFT therapeutic journey, so expect and be prepared for them; (2) Collaborate with patients to develop a formulation around the functions of the fears and their origin; (3) Use de‐shaming psychoeducation and guided discovery around the definition of compassion (often compassion is misunderstood to be soft, weak or indulgent); (4) Address fears through experiential practices such as method acting, imagery, chair‐work and compassionate letter‐writing; (5) Support the modelling of compassionate relationships with the therapeutic relationship.

Although some qualitative data exist examining fears, blocks and resistances to compassion and how therapists overcome these (Steindl et al., 2022), to this date, and to the best of our knowledge, there are only three other relevant studies that make use of the qualitative ‘Delphi’ technique in the context of compassion to iteratively delve into therapists' knowledge and experience. Liddell et al. (2017) investigated the main competencies of the CFT therapist using the Delphi method, identifying areas of clinician competence (e.g. create safe‐space, build therapeutic alliance, demonstrate compassion qualities and de‐shame through conveying common‐humanity) when delivering CFT (Liddell et al., 2017). In that study, however, overcoming fears, blocks and resistances to compassion was not examined specifically and did not emerge as a theme. A Delphi study, by Armstrong et al. enquired into the meaning of compassion, in terms of its role in ethical reasoning in psychology (Armstrong et al., 2000). However, that study defined compassion as a moral virtue and defined as such, compassion lacks the dimension of motivation which is central to the CFT model. Finally, Durkin et al. (2020) focused on how researchers of compassion in hospital settings defined compassion, reaching consensus that compassion is a ‘virtuous response involving awareness of and participation in the suffering of another conveyed through action intended to reduce the suffering observed’. Hence, the present study is the first to use the Delphi method to examine how patients in therapy see compassion and then can be helped to come to a new understanding within the constructs of CFT and overcome resistances to develop compassion for themselves.

## METHOD

### Aims

The primary aim of this study is to examine whether the understanding of the term ‘compassion’ influences engagement with/or resistance to the model of CFT. Objectives are 1. To use the Delphi method to collect expertise from clinicians about their patient's perceptions of the word ‘compassion’, that is how do they and their patients', or audiences, define and understand compassion. 2. To establish whether the perceived meaning of the term compassion might pose a barrier to engagement with CFT. 3. If the answer to question 2 is yes, to identify what are the specific elements that generate this resistance. 4. To suggest possible ways of mitigating misconceptions and resistances.

The study uses the Delphi method which ‘provides a method for structuring communication in a way that allows a group of respondents to confront a complex problem and to reach consensus’ (Linstone & Turoff, 1975). The Delphi has been extensively used in various settings where informed decision making is necessary or when research questions are complex and underlined by multiple factors. Examples include psychological research and the review of psychological training objectives (Graham & Milne, 2003); health care research (Trevelyan & Robinson, 2015); and examining aspects of patient‐centred care in ICUs (Oczkowski et al., 2017). The Delphi was therefore chosen as the most suitable investigative technique because this specialist research question requires ‘informed judgement’ to be explored, (Ziglio & Adler, 1996).

### Procedure and participants

Ethical approval for the study was obtained from the Health, Psychology and Social Care REC at the University of Derby. Participants provided written consent to participate in remote Delphi sessions. At the ‘exploration’ stage (Linstone & Turoff, 1975; Ziglio & Adler, 1996), we established the need for the study by informally consulting with CFT practitioners. We defined as ‘expert’ for inclusion in the panel psychotherapists, clinical psychologists and/or academic researchers who had been instrumental in developing, evaluating and delivering CFT and training/supervising other practitioners in CFT. Specifically, inclusion criteria stipulated that to ensure CFT model fidelity, respondents are formally trained in CFT either directly by its founder, Professor Paul Gilbert, or by a trainer employed by the Compassionate Mind Foundation and have significant experience of delivering CFT through their practice, or additional expertise in developing CFT through academic research.

Overall, eighteen practitioners were identified, ten of whom also had active roles in academic research regarding CFT. The age of participants ranged from 35 to 70 years old and their years of CFT delivery varied from 5 to 15 years. Of the eighteen people (11 male, 7 female) approached directly via email, one declined due to workload and two were unresponsive. Of the fifteen people who initially consented to take part, ten (6 men, 4 women) completed the first open round of the study and nine (5 men, 4 women) completed the second confirmatory round. This sample was deemed acceptable, because size calculation in Delphi does not depend on statistical calculation, but on representativeness (Okoli & Pawlowski, 2004, p. 20). In previous literature 10 (Okoli & Pawlowski, 2004) or 8 responses (Hallowel & Gambatese, 2010) to the initial round of questions was considered acceptable. In addition, due to the increased homogeneity of the pool of respondents a smaller sample is appropriate (Skulmoski et al., 2007, p. 10).

Participants received via email an information sheet and consent form, followed by a short questionnaire of five open‐ended questions: Q1. ‘In your experience, have you found that the perceived meaning of the term “compassion” has led to resistance with engaging with compassion‐focused therapy (CFT)/Compassionate Mind Training (CMT)’? ‘Please note, “resistance” does not mean complete unwillingness or rejection. Please indicate ‘Yes’ or ‘No’ in the box below; if your answer is NO, please provide a brief explanation, and return this form to us without filling in the questions that follow’; ‘If you have answered YES to Q1 above, please elaborate’; Q2. ‘What are the most common meanings given to compassion?’; Q3. ‘What are the most common misconceptions of compassion?’; Q4. ‘What other factors do you see as relevant in the understanding of the term compassion and any resistances? Are gender, age, or occupation relevant?’; Q5. ‘In your opinion or experience, does the resistance caused by the understanding of the term ‘compassion’, persist even after you have provided specific information on how your practice defines the term?’.

After initial analysis of replies, 15 further multiple‐choice questions (scored according to ‘strongly disagree‐strongly agree’) were generated and were returned to the panel for clarification in a confirmatory round. At the end of that round the researchers decided to close the study and to not seek further iterations as it was evident from both rounds that there was great extent of agreement between respondents and, crucially, that there was stability, that is consistency in the replies between rounds.

## RESULTS

The outcomes of this study can be summarised as follows:

1. Resistance to compassion does occur in therapeutic settings and is connected to misconceptions around the meaning of compassion.

(1a) The meaning of compassion is commonly equated to pity, weakness and ‘softness’.

(1b) Patients tend to miss the courage and wisdom aspects of the CFT definition of compassion.

2 No single demographic characteristic seems to emerge as a factor influencing patients' definition of compassion. Instead, valuing competitiveness and de‐valuing compassion emerged as a factor.
3 Misconceptions or resistance to compassion persist after the CFT definition of the term has been given.

(3a) Communicating meaning through defining compassion is insufficient and exposure to examples and experiences of compassion is necessary to reach clarification.

4 Time emerged as an important factor in reaching an understanding of compassion as defined by CFT.

Respondents unanimously agreed that misconceptions regarding the meaning of compassion are a cause of resistance to the therapy. Respondents noted commonly attributed meanings were ‘pity’, ‘softness’ and ‘weakness’. Responses further suggest that these combined misconceptions result in devaluing compassion as pity and viewing compassion as connected to a down‐rank position (as in to be pitied), whilst softness and weakness tended to be seen as avoidance of accountability (one participant used the phrases ‘not taking responsibility’ and ‘being easy on oneself’ [R6]). Hence, returning to the definition above, it can be said that, when first introduced to CFT, patients tend to miss the courage and wisdom elements that form an integral part of the CFT definition. As one respondent put it, common misconceptions derive from ‘grasping the kindness dimension without noting the strength and courageousness of it’ [R1]. In addition, the alleviation of suffering is also not initially referred to by patients. One respondent emphasised that people ‘certainly don't link it to a standard definition of specifically engaging with suffering’ [R5]. This is an interesting finding, especially because recent research found that when compassion is considered in relation to other prosocial terms such as kindness, the primary differentiator of compassion is seriousness and intensity of suffering alleviated (Gilbert et al., 2019).

It was generally agreed that misconceptions of compassion are very common and occur irrespectively of demographic characteristics. Whilst most respondents did not mention age as a factor, there were three contradictory responses with two suggesting that younger people seem more resistant to compassion (‘They see it as a skill of older adulthood, as they see their grandparents as compassionate people' [R10]) and one respondent suggesting the opposite. However, when the question was put to the second confirmatory round, the consensus was that age does not play a role. Occupation was also not seen as affecting the perception of the term. The religious connotations of the term were picked up by two participants. One of them remarked that this may mean that people have compassion ‘more readily at the fore‐front of their mind’, but also carry ‘specific connotations’ [R6]. Most participants did not identify significant gender differences, stating that, both men and women can be resistant to the term due to misconceptions. Three participants felt that men were more resistant and that this was reflected in the reduced number who engage with therapy and research. In addition to demographic characteristics, participants identified competitiveness and insecure attachment history as factors which caused misconceptions and resistance. Both were associated with devaluing and misconceiving compassion in the first round and had strong consensus in the second round.

The first round, open‐ended question, of whether misconceptions persist after the CFT definition has been provided, also received high levels of agreement. Respondents volunteered information on why this happens, or how they deal with it in a professional context, and further interesting results were yielded. Responses indicated that using only psychoeducation—that is, the theoretical part of the therapy where the evolutionary origins of compassion are explained—was not sufficient for an understanding of compassion to be achieved. Respondents noted the necessity of exposure to examples of, both giving and receiving compassion and to the experiential side of it. One participant described their approach in eliciting this: ‘I create opportunities for it to arise and then point it out… “*that*—what you just did there—that is what we mean by compassion’ [R2]. Others noted how real‐life examples or visualisations help and the need for people to experience it to fully understand it. The following statements were put to the second round to assess agreement: ‘People do not usually get it completely if you only use psychoeducation’; ‘People get what compassion is when they experience some form of it’. There was strong agreement on both statements, with one outlier response being ‘somewhat agree’.

Another finding related to the duration of therapy. Specifically, the initial round suggested a single, ‘enlightening’, moment whereby resistances fade away as soon as understanding of the meaning of compassion is reached, and in contrast others suggested it is a process that takes time ‘it takes time to properly appreciate the depth of understanding compassion’ [R4]. One participant noted that this process was not always linear: ‘The patient can go back to previous beliefs about compassion. But as you progress through the program and therapy you start to get more in touch with the real struggle and pain there can be some avoidance or fears that can come up later down the track. Which can mean coming back to the definition of compassion’ [R9]. Another respondent commented: ‘I think people might change the “meaning” [according to the CFT model] but then experience very visceral reactions to compassion practices and the negative “meaning” might return (e.g. it made me feel vulnerable/weak‐ even if I don't believe it to be so)’ [R6]. Further clarification in round two showed exceptionally strong agreement (no outlier responses and the most ‘Strongly agree’ indications) with the statement: ‘It takes time to properly appreciate the depth of compassion and understand it fully’.

## DISCUSSION

This study confirms that resistance to compassion is commonly experienced and like the findings of Steindl et al. (2022), practitioners should expect and be prepared for these resistances. The study also found that resistance often derives from misconceptions around the meaning of compassion. More specifically, respondents in this study have noticed the association of compassion with ‘pity’ and ‘weakness’ and generally terms that imply low‐rank social positions. In turn, this diminishes engagement with compassion as it is viewed negatively. This finding is again consistent with those of Steindl et al. (2022) who found misconceptions around compassion being seen as soft, weak or indulgent. This is significant because to associate compassion with weakness and low‐rank position misconstrues how CFT understands the term and undermines its therapeutic potential. It is only when compassion is understood in its appropriate context—of motivation and competencies, the cognitive ability to understand what is required to be compassionate, and the courage to act on that understanding—that it can lead to benefits for the individual. As Catarino et al. (2014) have shown, when the desire to be compassionate and helpful is related to low‐rank appeasing and submissive behaviour, what they term ‘submissive compassion’ rather than genuine compassion, it is associated with ‘shame‐based caring, depression, anxiety and stress’(Catarino et al., 2014).

No single demographic characteristic was strongly identified as a factor in the likelihood of misinterpreting compassion, suggesting misconceptions of compassion are pervasive. Rather, strongly valuing competitiveness was identified by respondents as a key characteristic in seeing compassion in terms of low‐rank positions and devaluing compassion. This is consistent with the findings of Basran et al. (2019) who found competitiveness to be associated with greater fears and resistances to compassion.

How we feel about compassion and how, in turn, it makes us feel are important components of its meaning. Definitions and descriptions, which engage our cognitive abilities seem to be inadequate by themselves. In this respect, the most consequential outcome of this study is that the experiential part of the therapy and the use of worked examples is very important in instilling an understanding of compassion. Significantly, in a previous study of how compassion is perceived by people experiencing depression and anxiety, the majority of patients drew on their experiences of being compassionate towards others and offered examples of when they had been compassionate (Pauley & McPherson, 2010, p. 134). This again also complements the findings of Steindl et al. (2022) who found that experiential practices such as method acting, imagery, chair‐work and compassionate letter‐writing were essential for overcoming fears of compassion.

Understanding and applying compassion is a process that takes time not only due it being a multifaceted term but also because the process entails amendment of common misconceptions and assimilation of new knowledge. In the case of CFT, the new knowledge that is to be assimilated is that compassion requires strength, wisdom and courage and that it entails motivation and competencies. This process may not be linear or straightforward. As noted by respondents, even after a renewed understanding of compassion has been reached, people may return to previous misconceptions. The aim of CFT, and of compassion research in general, is to cultivate compassion. This would not be achievable if meaning disparity was an insurmountable barrier. As one respondent summarised: ‘It often takes several sessions, but almost everyone gets there eventually’ [R1].

### Limitations

The study is limited due to its small sample size, as it was the expertise and fidelity to the CFT model plus the depth of data that was prioritised rather than breadth. Whilst this study sought the opinions of those delivering CFT, it is advisable that a more extended future study also involves responses from patients and members of the public who have had experience of receiving CFT.

## CONCLUSION AND IMPLICATIONS

The study offers some important implications for CFT practitioners: (1) the CFT practitioner can both expect and be prepared for resistances to compassion due to misconceptions about compassion being associated with pity and low social rank positions, missing the courage and wisdom elements of the CFT definition of compassion; (2) psychoeducation and cognitive definitions of compassion are insufficient, exposure to examples and experiential practices of compassion are necessary to reach clarification; (3) no single demographic characteristic emerged as a factor influencing patients' definition of compassion. Instead, valuing competitiveness and de‐valuing compassion emerged as a factor for practitioners to address; (4) Misconceptions or resistance to compassion often persist after the CFT definition of the term has been given, practitioners should be prepared for a non‐linear progression; and (5) It takes time to reach an understanding of compassion as defined by CFT but most patients reach an understanding in the end.

## AUTHOR CONTRIBUTIONS

**Kirsten McEwan:** Formal analysis; investigation; methodology; supervision; writing – review and editing. **Lina Minou:** Conceptualization; formal analysis; investigation; project administration; writing – original draft.

## CONFLICT OF INTEREST

None.

## Data Availability

Anonymised data are available for the purpose of research and audit on request by the corresponding author.
